# Frederick Stearns & Co.’s New Vaccine Building

**Published:** 1903-12

**Authors:** 


### Abstracts and Selections.

### Frederick Stearns & Co.'s New Vaccine Building.

The new building for housing the calves and performing the operations necessary to the propagation of vaccine is almost finished. It is on the site of the former one, which was razed to make room for it. A fuller description will be given later, but a statement of a few of its many advantages and conveniences may be of interest at this time.

This building is entirely of brick, has but one outside entrance and no windows whatever, being lighted by skylights of the most modern and approved form, fitted with prismatic glass, which diffuses a very strong light without allowing direct rays of sunshine to enter. Ventilation is had by means of powerful patent ventilators and electric fans. Thus the dust from outside sources is absolutely excluded, which is an important item in the production of pure vaccine. The building is heated by steam, lighted by electricity and provided with hot and cold water throughout.

The large incubating room is equipped with iron stalls provided with small racks which the calves stand on. This raises the animals from the floor so that they do not stand in their own excretions even for a moment. The walls of this room are of pressed brick, enameled white, and the ceiling is arched or "coved," doing away with corners. The floor is of concrete, drained by a number of sewers. There is no wood in this room at all, even the racks on which the animals stand being iron, and these racks are scrubbed, sunned and sterilized daily, there being two complete sets. The room is cleaned daily by flushing with a hose.

The cleaning and sterilizing room adjoins the operating room. Both of these are practically porcelain lined, the walls being of glazed white tile with all joints enameled, making a continuous snow-white waterproof surface as smooth as glass. The ceilings are coved here also, doing away with corners. The light is from skylights and the ventilation through flues. Neither of these rooms has an outside door or window. The sterilizing room is provided with electric clippers, by means of which the calves from the quarantine barn are very closely clipped; then the calves are scrubbed in a hot antiseptic bath in the vats with which this room is provided. After being thus thoroughly cleansed they are shaved over the area to which the vaccine is applied. Then, being transferred to the next room, they are vaccinated and placed in the incubating room first described and fed on sterilized milk during the progress of the vaccinia which has been induced. It should, perhaps, be stated here that when the calves are first received from the farm they are placed in a quarantine station devoted entirely to their use, and are kept here for some time under the inspection of our head veterinarian, to demonstrate their entire healthiness, before being vaccinated.

The operating room is as clean and aseptic as that of a modern hospital. It is provided with all necessary apparatus, and, like the operating room of the antitoxin laboratory, is considered without an equal.

In this building also, but entirely separate from the vaccine department and not communicating with it in any way either by door or window, are the large injecting and bleeding rooms of the antitoxin laboratory. In the first of these rooms horses are injected with the toxins. The walls of this room are of pressed brick covered with white, waterproof enamel, and having coved ceiling and concrete floor. The operating room, which communicates with this, has walls of white glazed tile, exactly like those in the vaccine operating room, with arched ceiling and cement floor. The instruments are kept in plate glass cases, the antiseptic solutions, etc., on plate glass shelves—in fact, the arrangements are very similar to those of a hospital, as has been stated.

Taken as a whole, nothing has been left undone that science could suggest and money could procure that would tend to make this portion of our antitoxin and vaccine establishment ideally perfect.

